# Adapting the WHO ANC digital module for the NAMAI study: Formative research to inform implementation science interventions for enhanced quality service delivery following the WHO SMART guidelines approach

**DOI:** 10.1371/journal.pdig.0000910

**Published:** 2025-06-23

**Authors:** Nachela Chelwa, Bernard R. Ngabo, Muyereka Nyirenda, Musange F. Sabine, María Barreix, Tigest Tamrat, Natasha Okpara, Chifundo Phiri, Nathalie K. Murindahabi, David Nzeyimana, Tobias Makai, Gilbert Uwayezu, Gladys Yabalwazi, Mwamba Kangwa, Rosemary K. Muliokela, Hedieh Mehrtash, Caren Chizuni, Vincent Mutabazi, Felix Sayinzoga, Michael T. Mbizvo, Maurice Bucagu, Özge Tunçalp

**Affiliations:** 1 Population Council, Lusaka, Zambia; 2 School of Public Health, College of Medicine and Health Sciences, University of Rwanda, Kigali, Rwanda; 3 Zambia Country Office, World Health Organization, Lusaka, Zambia; 4 UNDP/UNFPA/UNICEF/World Bank Special Program of Research, Development and Research Training in Human Reproduction (HRP), Department of Sexual and Reproductive Health and Research (SRH), World Health Organization, Geneva, Switzerland; 5 Blue code Systems, Lusaka, Zambia; 6 1000Hills Solutions, Kigali, Rwanda; 7 Ministry of Health Zambia, Lusaka, Zambia; 8 Rwanda Country Office, World Health Organization, Kigali, Rwanda; 9 Rwanda Biomedical Center, Kigali, Rwanda; Center for Primary Care and Public Health: Unisante, SWITZERLAND

## Abstract

The Ministries of Health in Zambia and Rwanda have adapted and validated their national antenatal care (ANC) guidelines in line with WHO 2016 recommendations. Both countries conducted implementation research, composed of five implementation strategies to support the adapted ANC package service delivery. One implementation strategy deploys a digital module, a point of service digital tool that encompasses clinical decision support and person-centric record management to support health workers in implementing the adapted ANC packages. The formative phase of the study, included countries’ adaptation of the WHO digital ANC module to their contexts in three steps: (i) the reference module was tailored to create Rwanda and Zambia ANC digital modules and training materials; (ii) health workers were trained to use the module and provide feedback; (iii) country research teams conducted qualitative assessments to understand the health worker experience using the adapted ANC module. ANC health workers at selected facilities completed a three-day training on the use of the module. Qualitative methods were used to understand health worker’s perspectives on the module’s use for service provision and feedback for its refinement. The three major themes emerged: i) experiences using digital interventions in the health profession; ii) strengths and challenges related to the use of digital interventions; iii) considerations for improving the use of digital interventions within health systems. Rwanda and Zambia ANC modules were modified to improve their use for ANC services delivery. Initial testing led to the identification and fixing of bugs in the system. The module was updated to include dashboards to support facility-based monitoring of ANC indicators. Training materials were also improved based on feedback from interviews of health workers. The iterative process in developing country-adapted digital ANC modules is key to their deployments for routine use and a key proof of concept for the WHO SMART guideline approach.

## Author summary

We developed a digital tool and its related training material for health workers to use when providing care for pregnant women in Rwanda and Zambia. Health workers who treat pregnant women were trained and asked questions regarding the digital tool, to make sure it would be the most helpful to them. We analyzed their comments and made changes to the digital tool, and training material. The subsequent phase of this study will assess if the digital tool improves the health care that pregnant women receive.

## Introduction

Countries in sub-Saharan Africa (SSA), including Zambia and Rwanda continue to record high pregnancy-related morbidity and mortality rates [1]. According to World Health Organization (WHO), SSA “alone accounted for approximately 70% of global maternal deaths into 2023” [2]. Across the sexual and reproductive health continuum, antenatal care (ANC) services are a crucial point of introduction for pregnant women into the health system [3,4]. To improve the quality of ANC, the WHO has produced recommendations on ANC for a positive pregnancy experience describing timely evidence-based interventions to be provided and which can improve maternal and fetal outcomes [3,5]. Nonetheless, numerous barriers remain to implementing these recommendations, adapted to local contexts, and at the point of care [6,7].

**Fig 1:**
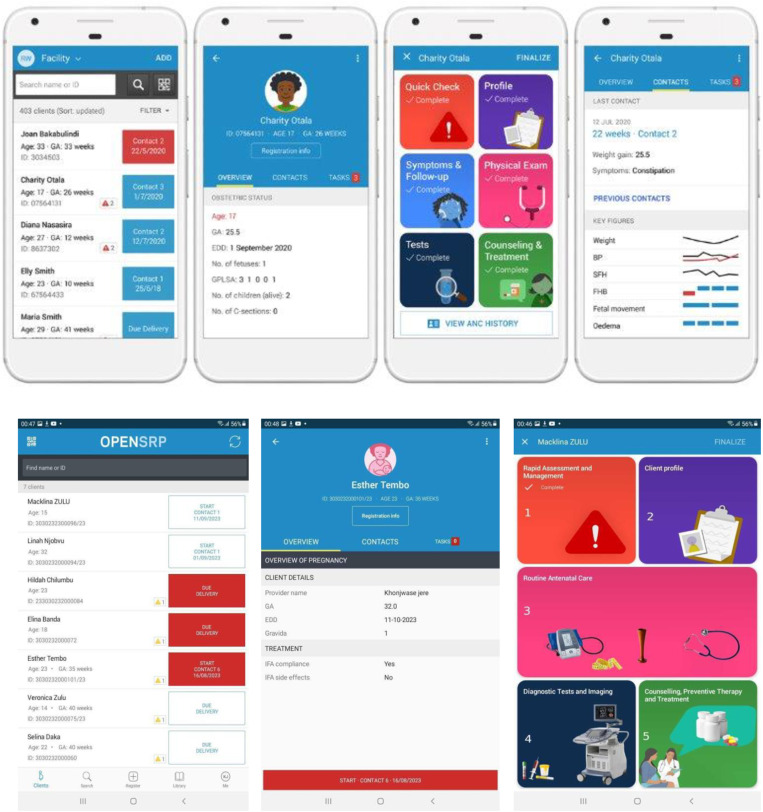
Screenshot of Rwandan and Zambian ANC digital modules (respectively).

Since 2018, the Ministries of Health (MOHs) in Zambia and Rwanda, with support from WHO, have adapted and validated their national ANC guidelines, in line with the WHO 2016 ANC recommendations [8]. Subsequent to those efforts, both countries conducted an implementation research study, *New Antenatal Model in Africa and India (NAMAI) study: an implementation research study to improve antenatal care using WHO recommendations*, to understand what it takes to implement the country-adapted ANC policies and related intervention package of ANC services [9]. One of the study implementation strategies leverages the WHO digital ANC module (or reference module), a smartphone-based software that encompasses a clinical decision support and management, tracking and patient-centric record tool, based on WHO guideline-derived algorithms, which aims to support health workers in providing the country-adapted ANC service packages [10,11]. The reference module’s development has been previously documented [12] and is in line with the WHO’s SMART guidelines approach [13]. As part of its contextualization, the reference module was modified based on country-adapted versions of the ANC Digital Adaptation Kit (DAK) [14]. DAKs represent software neutral requirements documentation which distill WHO recommendations into a format that can more easily be integrated within any digital system [10]. In this case, country-adapted versions of the DAK were applied to guide the customization of the reference module to the Rwanda and Zambian contexts [13,15]. The country-adapted digital modules were designed for health workers to employ at the point of care, while pregnant women are receiving services, guiding the worker through the adapted national ANC service package, ensuring that all relevant interventions (e.g.,: history taking, laboratory tests, physical exam, counselling, suggested diagnoses and treatments) are provided. Please see Fig 1 for screenshots of the Rwandan and Zambian ANC digital modules.

**Fig 2:**
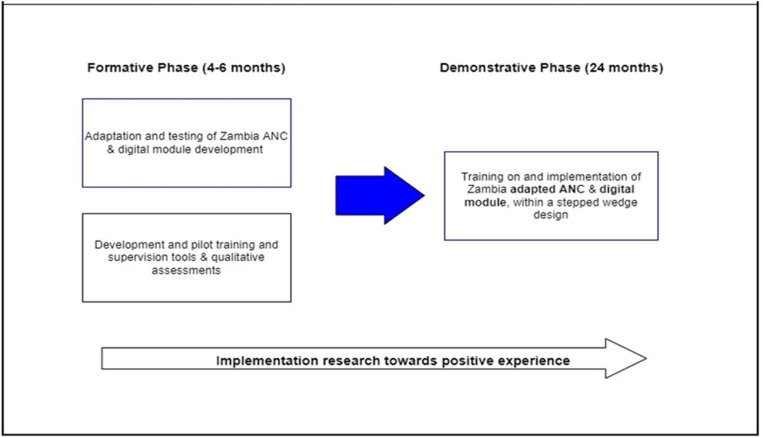
Outline of methodological steps and expected results.

The NAMAI study had two phases. The formative phase focused on adapting the reference module and related materials to each country’s context. The subsequent demonstration phase consisted of a mixed methods stepped-wedge cluster randomized implementation trial, described elsewhere [9]. Fig 2 illustrates the key components which were implemented during the formative phase, culminating in the development of a digital intervention for the demonstration phase.

Prior to the formative phase, the reference module was mapped to the digital landscapes in both countries and plans made for integration to existing data aggregation, and other relevant, digital platforms [11]. Additionally, workshops were held with relevant stakeholders (from national, regional and facility levels) to revise the WHO ANC DAK content, which details the data elements, decision support, and indicators underlaying the reference module, to the Rwandan and Zambian contexts. This standardized process, ensured that all digital systems based on the DAK would reflect updated national ANC guidelines. The country-specific DAKs served as the basis for the adapted digital modules detailed in this manuscript.

The formative phase objectives were to: 1) adapt the reference module to the country-specific ANC package and local healthcare delivery context, 2) identify bugs and errors in the clinical algorithms in the country-adapted ANC digital module, 3) gather general feedback regarding the usability of the country-adapted ANC digital module and components for improvement, and 4) adapt and test training materials for the country-adapted ANC digital module in Zambia and Rwanda [16]. This paper describes the experience and findings from the formative phase of the NAMAI study in both countries.

## Materials and methods

Formative phase field activities were jointly conducted by the country research teams composed of the MoHs, in Rwanda this also included the Rwanda Biomedical Centre (RBC), the local implementation research partners (University of Rwanda School of Public Health and Population Council – Zambia), technical partners (1000Hills Solutions and BlueCode Systems), and the WHO (country, regional and headquarters offices). Technical partners were supported by Ona Systems Inc., a Kenya based Information Communication Technology (ICT) firm which developed the reference module.

This formative study phase consisted of the following steps: adaptation and testing the country-adapted ANC digital modules, end-user training, and qualitative assessment. Firstly, the country-customized ANC DAKs were reviewed to inform the customization requirements for tailoring the reference module for Rwandan and Zambian ANC service packages [11]. To do this, the WHO ANC DAK was previously contextualized to the national ANC service delivery package during a mapping exercise and stakeholder consultations [11]. Country-adapted versions of the reference module were developed, reviewed and tested by study teams. Additionally, training materials for the reference module, comprising of a user and facilitators guide, training presentations and learning assessment tools, were tailored to the country adapted digital modules [16]. Subsequently, health workers at selected health facilities were trained on the country adapted digital module using these training materials. Finally, the country research teams conducted focus group discussions (FGDs) and in-depth interviews (IDIs) to further understand the health workers’ experience deploying the ANC digital module.

### Adaptation and testing the ANC digital module

A series of stakeholder meetings were held to align the ANC digital module requirements with DAK components, including gaining consensus on how to integrate the module with existing ANC tools and systems such as Health Management Information Systems (HMIS), the Zambian SmartCare system and Rwandan Electronic Medical Records (EMR). Using the outputs from the customization of the ANC DAKs, country teams in Rwanda and Zambia were tasked with translating the reference module [12] to a national version of the module using updated ANC guidelines and clinical workflows. Technology partners in each country participated in a two-week knowledge transfer and virtual training sessions conducted by a technology company, tasked with coordinating the customization process for the reference module. A series of meetings were held to understand the reference module’s requirements in relation to the DAK components, including planning the integration of the country-adapted modules into existing ANC tools and systems. Additionally, country teams conducted visits to health facilities to validate module requirements regarding ANC workflows as well as mapping content across the reference module, the DAK and existing facility registers. Based on these efforts, technical partners built and tested multiple iterations of the Rwanda and Zambia ANC digital modules. Decisions on further modifications and inclusions into the country-adapted modules were reached through consensus by relevant stakeholders based on the adapted-ANC DAKs, national protocols, and guidelines. In Rwanda, customization included a translation of the content into French to enabling access to health workers who are not fluent in English.

Additionally, in Zambia, initial testing was conducted at an urban high volume health facility (Kalingalinga Health Centre) in Lusaka during which four field visits were carried out by the research team to collect preliminary feedback on the Zambian ANC digital module prior to conducting the qualitative assessment.

### End-user training on digital tool

Formative phase facilities in both countries were selected for having ANC coverage and staffing levels comparable to those of the demonstration phase facilities. In Zambia, the formative phase was conducted in three Zonal Health Facilities (ZHFs) in Eastern province: Mchini Health post in Chipata district, Nsadzu Zonal Health Center (ZHC) in Chadiza district and Mphangwe Rural Health Centre (RHC) in Katete district. A total of 12 health workers were trained on the Zambian ANC digital module, which included three district officers and nine health workers. In Rwanda, the study was carried out in six primary health facilities in Bugesera district: Gashora, Gakurazo, Mayange, Juru, Nyamata, and Ruhuha. Two health workers (nurses and/or midwives) working in the ANC departments of the six facilities, attended the ANC digital module training.

Selected health workers participated in a 3-day training on the use of the country-adapted module; the trainings took place in January, March and June 2022 (Zambia) and February 2022 (Rwanda). Trainings in Zambia were conducted at each formative phase facility, separately. The first day was used to explain the purpose, content, and use of the module. The second day consisted of health workers carrying out mock or practice exercises. Structured training sessions were conducted on the use and navigation through the country-adapted module. This was followed by feedback sessions on the areas requiring improvement and integration into the module and training materials. The research team then assisted the health workers in employing the Zambian ANC digital module for the first five to six pregnant women attending their first ANC contact. ANC health workers provided care to pregnant women in pairs; while one health worker employed traditional data collection methods (paper registry/patient file), the other completed the Zambian l ANC module using a tablet.

In Rwanda, the training was held at the University of Rwanda, Kicukiro campus in Kigali. Over the three days, the participants were introduced to the overall purpose of the research project, the objective, content, and use of the Rwandan ANC digital module. A short introduction to the use of digital devices was also conducted to ensure that participants were able to deploy them. The health workers then participated in mock ANC service provision role playing using the Rwandan ANC digital module. After feedback from the training was incorporated to an updated version of the Rwandan ANC digital module, participants took the tablets back to their primary health facilities and were able to use them while providing ANC services for the following six-week period.

### Qualitative assessments

#### Participant selection and study setting.

FGDs and IDIs were used to understand the health workers’ and supervisors’/head of health centre perspectives on using the Rwandan/Zambian ANC digital module for service provision. In Zambia, convenience sampling was employed to select nine personnel, they included six health workers and three supervisors for FGDs across 3 health facilities. In Rwanda, the study adopted a purposive sample of selected participants. Two health workers, working in the ANC departments, from the six facilities (12 total), as well as their supervisors (six heads of health centre), participated. Interview were conducted by trained qualitative research experts from the local study implementation research partners: Population Council - Zambia, and the University of Rwanda. FGDs and IDIs were structured, using interview guides developed as part of the protocol [9]. Participants were invited to take part in a FGD or IDI at the end of the training and lasted between 60- and 90- minutes. Participants across both countries included nurses, nurse midwives or medical officers who were responsible for providing ANC or supervising those providing ANC in the study facilities. Their ages ranged from 25 to 45 years old.

In Zambia, three FGDs were held with two health workers across each facility, and three IDIs with each of the supervisors across the three facilities. On the final day of training, the trained health workers participated in an FGD at the end of training to gather feedback on the Zambian ANC digital module’s design, structure and content. Additionally, IDIs gauged the facility’s supervisors’ feedback not only on the Zambian ANC digital module but also on the training material, technical and supervisory mechanisms that would be required for supporting its deployment during the demonstration phase. Based on the FGDs and IDIs results, the Zambia ANC digital module was modified, and an updated version was presented during a full day workshop to the study’s Technical Advisory Group (TAG), comprising representatives of MoH headquarters and provincial staff, Midwives Association of Zambia (MAZ), Zambia Association of Gynaecologists and Obstetricians (ZAGO), WHO, Population Council and BlueCode staff. Feedback received was used to further refine the module.

In Rwanda, an FGD composed of 12 participants was held on the final day of training. Based on the FGD results, the first version of the Rwandan ANC digital module was modified, and a second version was developed and delivered to the same ANC health workers in March, 2022, for use in their respective health facilities. This module version was used in the six formative facilities for a month and a half of testing the country-adapted digital ANC module; this entailed using the module on an ad-hoc basis on the days that ANC was provided at the facility. Afterwards, two health facility staff, one of the trained ANC health workers and the head of the health centre, from each of the six health facilities were invited to a one-day feedback session in May, 2022. After this session, two FGDs were conducted, one with six ANC health workers and the second with the six heads of health centres to gather final thoughts, challenges, recommendations and considerations for the deployment of the digital module that led to the creation of the version of the Rwandan ANC digital module for deployment during the demonstration phase.

### Data analysis

In Rwanda, FGDs were conducted in Kinyarwanda, audio-recorded, transcribed in verbatim, cleaned, and summary notes and quotes translated into English. In Zambia, FGDs and IDIs were conducted in English, audio recorded, transcribed, and reviewed against field notes and the audio file for accuracy in transcription. In both countries, a qualitative research expert performed quality checks of the transcribed and translated files so that they accurately capture the information shared by respondents in their context.

For both countries, a thematic analysis was conducted and all five steps of qualitative-data analysis (reading, interpreting, coding, reducing, and displaying) were performed to ensure consistency within the data [17]. Upon completion of transcription and translation and to respond to the study objectives, a codebook was developed which frames the key themes from the qualitative data. Proposed codes are related to the overall experience in using the country-adapted digital ANC modules: usefulness, training received, the effect of digital ANC on quality of routine data, supervision needed, organization of health services, challenges, and suggestions to improve the ANC modules. Translated files were imported into ATLAS.ti version 9.1.5 software (Rwanda) and Nvivo 12 (Zambia) for data analysis.

Ethical approvals were obtained from the Rwanda National Ethics Committee (RNEC) (No.650/RNEC/2021), ERES Converge (Zambia - 2020-Sep-009), Population Council Institutional Review Board (Protocol 980), and WHO Ethics Review Committee (A66008) before data collection. Scientific review approval from the Rwanda Ministry of Health Research Committee was also granted (NHRC/2021/PROT/009). Administrative approvals were also received from the Zambian National Health Research Authority (NHRA00001/23/10/2020), and Ministry of Health. All participants were provided with study information and gave written informed consent to participate and to record the FGDs/IDIs before their participation. FGDs/IDIs were held in a private space to ensure confidentiality. All participants were given pseudonyms to ensure anonymity and names, and any other personal information were not recorded on written or audio files.

## Results

The results of the FGDs and IDIs were merged into three major themes, combining both ANC health workers’ and their supervisors’/head of health centre’s perceptions: i) experiences using digital interventions in the health profession; ii) strengths and challenges related to the use of the digital intervention; iii) considerations for improving the use of digital interventions within health systems. Within the themes, 11 subthemes were generated, all summarized in Table 1.

**Table 1:** Themes and subthemes of the formative phase.

Theme: Experiences using digital interventions within health systems
Subthemes •* Low level of knowledge and skills using digital technology (Rwanda)* •* Importance of training on the module’s use (Zambia)* •* Limited internet access (Zambia)*
**Theme: Strengths and challenges related to the use and design of ANC module**
Subthemes - strengths •* Ease of use of the module (both)* •* Module ensures completeness of data and provision of full package of ANC services (both)* •* Longevity of the data within digital systems (Rwanda)*
Subthemes - challenges •* Module delays and crashes, and limitations (Rwanda)* •* Module content – omissions regarding HIV services (Zambia)* •* Sequencing of patient flow (Zambia)*
**Theme: Considerations for improving the use of ANC module for the provision of care**
Subthemes •* Human resources constraints (both)* •* Avoiding double reporting and time burden (both)*

### Experiences using digital interventions within health systems

- ***Low level of knowledge and skills using digital technology (Rwanda)***

Generally, the nurses and midwives reported a low level of knowledge and skills in using digital tools in their profession. Some of them mentioned the lack of time to get acquainted with electronic devices due to their workloads. Others reported that they were not familiar with electronic devices and other digital tools in delivering medical services because they went to school when digital technology was not available. One health worker, who is a recent graduate expressed the following: *“As it is being frequently said, our use of digital technology is still poor. I am a fresh graduate. I studied at a time when digital technology is available, but I may have a colleague who went to school before we had access to digital technology. Therefore, it is challenging for that person to adapt quickly.” (FGD participant)*

Related to limited digital knowledge and skills, some participants mentioned that age and mindset are important factors for the uptake of the ANC digital module. The following quote illustrates this point: *“…. Also, the age of the nurse/midwife may be a challenge because they are not used to digital technology and they must do something completely new; so, using a laptop or tablet is a challenge to them. Some health workers are very old that they are challenged by digital technology, and anything to do with it is a challenge. Moreover, there are others with a poor mindset who think that digital technology is only for the young people”* (FGD participant).

- ***Importance of training on the module’s use (Zambia)***

Health workers gave insights into training/orientation to the ANC module. They reported that the training was useful and reminded them of components sometimes forgotten during ANC service provision. “*I learnt a lot, such as giving calcium and how everything should be done to an expectant mother. It’s so nice that we are able to use a digital device.”* (FGD participant). Additionally, they noted that training was short and very focused on the first ANC contact, and more is needed regarding additional contacts. “*The training was okay, though, too short for someone to understand everything from the registration to the end”* (FGD participant).

- ***Limited internet access (Zambia)***

Health workers were concerned regarding what would happen to the data gathered if there was no internet, because the ANC module requires internet to synchronise data at the end of a contact. Given that most facilities where testing occurred were rural, the unstable internet could affect how often the data is uploaded. “*It’s good that the app can be used while offline, but the only challenge may be on the syncing of data because we will need to find where there is good network to sync”* (FGD participant).

### Strengths and challenges related to the use and design of the digital ANC module

- ***Ease of use of the module (both)***

Health workers reported that the module was easy and exciting to use. They particularly appreciated the decision support mechanism which would flag services missed or highlight the need to refer or observe certain health conditions. “*I have not disliked anything, you cannot dislike something that makes your job easier, the manual registers are quick to use but don’t have everything. The module has everything, and it creates a good relationship between the client and health workers”* (IDI participant, Zambia).

When health workers tested the ANC digital module among women reporting for their first ANC contact, health workers noted that the ANC digitalmodule simplified their work and made it easier to provide care to the women. They observed that it was closely aligned to how services were provided at the facility. Similarly, a FGD participant in Rwanda stated “*compared to the registers we have to fill, the digital module is easier to use, and it accelerates the process. We are directed with the digital module so that even the information we forgot to look or ask for is there”.*

- ***Module ensures completeness of data and provision of full package of ANC services (both)***

Participants described the Rwandan ANC digital module as a complete tool that contains all the relevant data about the pregnant woman. They reported ease in retrieving pregnant women’s data when needed in comparison to having to go through the registers. They also appreciated being able to track the pregnant woman’s history and have all the data, such as lab tests in one place. This was emphasized by the heads of health centres who said that this will improve their motivation in following up pregnant women, with the ANC digital module, entered information cannot be lost, as illustrated in the following quote: *“…the first thing that got us excited is that when we have received a client, we cannot lose their data. We just have to type the client’s details in the tablet and can track that a mother attended the previous visit and see the trend of her visits, which is different from the way it was being done previously, where a provider had to search through the registers. So, this tool is so important, and it will help us in the follow-up of the pregnant women…”* (FGD participant, Rwanda).

Health workers also stressed the importance of having to enter the data into the system when registering the pregnant woman. If they accidentally omit any information while filling out the ANC digital module, it would prompt them to fill it in. Similarly, the ANC digital module made sure that essential delivery steps were not skipped and that women received the complete package of services in accordance with national guidelines. Participants appreciated the decision prompts and alerts for alarming readings and for fields not completed which would often end up blank in the paper registers. “*For the manual register, if certain things are not done, it is okay. Whereas the digital one will require health workers to do every step and at the national level, they will be able to see what we are not doing.”* (IDI participant, Zambia).

- ***Longevity of the data within digital systems (Rwanda)***

The ANC digital module allows health workers and their head of health centre to store client’s data for an extended period which can facilitate future analysis and use for the woman’s care. Some heads of health centre reported that this is particularly important because registers may get damaged or lost making it difficult to access data when it is needed. One of them said, “*the data from mothers who attend the ANC services is well kept and can be kept for a long time such that whenever anyone with access to the tool wants to check it, will find it, which is different from using the registers because sometimes they would be damaged and or go missing and when someone needs the mothers’ data, they won’t be able to get it.”* (Rwanda FGD participant).

- ***Module delays and crashes, and limitations (Rwanda)***

Participants reported that some ANC digital module sections took too long to load on the tablet, which slowed down the registration process. Additionally, the system occasionally crashed during registration which frustrated users and delayed the delivery of ANC services. A health worker described it, *“*…*sometimes, the page becomes unresponsive, and you have to get back to the start. Sometimes it used to happen to me and when I am tired, I felt angry and closed the system… When I attempted to go faster and force the system, the page became unresponsive and as I continued, the system crashed down and went back to the login page. The target I had fixed for myself was not achieved”* (FGD participant, Rwanda). Most health workers also recommended that the module allow the user to skip sections where the information is unavailable while registering the client in order to go on to the next section without having to wait for the previous section to be completed.

- ***Module content – omissions regarding HIV services (Zambia)***

Despite the positive experience using the ANC digital module, some health workers felt that it omitted certain services which were necessary for provision of quality ANC. This was specifically true for the HIV component of the Zambian ANC digital module, which health workers suggested could be enriched or provide more areas of documenting patient history by including HIV recency test, details on pre-exposure prophylaxis (PrEP), drug adherence education and viral load testing, personal hygiene during pregnancy, and accounting for women who would have changed health facilities after initial registration at a different facility. *“The only part of HIV was just the testing and if the mother is also on ART that is all, but what about mothers who would want PrEP and those mothers who are already HIV positive. What education or further information are we giving them? Because if they’re on an ART they also need information on drug adherence. They need that and they need to be coming so that their viral load can be checked and all that. So, I think that should also be included, so more content on [information education and communication]”* (FGD participant, Zambia).

- ***Sequencing of patient flow (Zambia)***

Health workers also noted that the sequencing/flow of the ANC digital module content was not systematic, making it difficult for them to ensure that all the service components were provided. They wanted the content to be arranged sequentially, and allocated numbers following the order in which procedures are arranged, rather than being flexible for a health worker to move between different service components. Participants also requested that the additional counselling provided to women be included in the ANC digital module. “*The [content is] not in order and should be arranged starting with the Rapid Assessment and Management (RAM), Client Profile, Routine ANC, Diagnostic Tests and Counselling and Treatment*” (FGD participant, Zambia).

### Considerations for improving the use of the ANC Module for provision of care

- ***Human resources constraints (both)***

Across study sites, supervisors/heads of health centres mentioned the limited number of personnel as a common challenge making it difficult to allocate staff to the different departments. This has an impact on the number of health workers available in the ANC department and the use of the ANC digital module. One of the head of facility said, *“…Only the availability of 2 [health workers] in ANC was challenging. Using the ANC digital tool when registering the client was so difficult for the [health worker] who worked in ANC during the day that she had to fill the data into the system throughout the night and that was quite overwhelming for her. Despite the system’s overall good design, it is difficult to enroll everyone into the system due to the shortage of health workers”* (FGD participant, Rwanda). In Zambia, health workers also noted the constraint in staffing, even when there were two health workers, “…*Challenges may arise on staffing, they are just two at the facility and sometimes they work alone in ANC …” (FGD participant, Zambia).* This was also expressed by health workers who had to handle the ANC digital module while alone in the ANC service ward. This resulted in increased workload and affected the quality of services delivered to clients, as expressed by a participant, *“We have a challenge with insufficient [health workers]; working alone in the ANC department is almost impossible, we can attest to that but because we must do it, it works despite challenges. We get quality assurance problems, and the mothers are complaining because when we provide services and delay, they do not even recognize what we have done even though we are exhausted. They only see that we have done nothing because we delayed them. Imagine receiving a pregnant mother at 8 am and sending her back at 4 pm. Even though we worked hard and got tired, I felt guilty for keeping women for so long when they were hungry. Without sufficient personnel, we cannot effectively use this digital module as one person while performing other tasks ….”* (FGD participant, Rwanda).

- ***Avoiding double reporting and time burden (both)***

The process of simultaneously filling the ANC digital module and the paper registers was reported as a significant challenge. Health workers found the process cumbersome because they must fill out the same information in various sources: the ANC digital module, the paper registers, patient files or ANC card which increased their workload, caused delays and affected ANC service delivery. One of the health workers stated, *“It is so burdensome; using the registers which are so many and then, this time to include entering data in the ANC digital module…We also entered the data at night, when we are on night duty or after finishing the work with physical examinations of the mothers…”* (FGD participant, Rwanda).

Some health workers expressed that they spent around 30 minutes with the ANC digital module compared to the average of about 10 minutes using the pregnant woman’s paper-based ANC card. Given all the details to be captured linked to the essential packages for ANC, health workers spend more time with each pregnant women to ensure that all the key fields are completed and that the essential investigations are undertaken. Health workers felt that these delays would affect how quickly each woman would be seen at the facility. One nurse-midwife stated, “*I think generally …, I can see a situation where women may take a bit longer to be at the facility, because one will need to input data on both, unless if the Ministry is saying they do away with the paper registers probably that can work, you will find that I need to input data on this tablet, I need to input data in the other ANC surveillance tablet, and then there are consent forms that clients need to sign, the tests that they need to undergo. So, I am foreseeing a point where clients may take a bit longer to be at the facility”* (IDI participant, Zambia).

In fact, to support health workers perform their duties effectively, participants recommended the use of the ANC digital module only, since it is easier to use and it records the same information as paper registers and patient files. As one participant stated, *“The delay may be because we were using the digital module coupled with filling the registers, otherwise I think it does not take long and the mother cannot be delayed…When we are two [health workers] in the consultation room, you can immediately inform your colleague and she/he enters the information into the system. The first time we used the system and filled in all the information we usually fill in but also include filling out the register it was an added workload but using the digital module alone does not take much time”* (FGD participant, Rwanda).

### Finalizing the digital intervention for the NAMAI study

Based on this process and findings, the Rwanda and Zambia ANC digital modules were modified to improve their use for ANC services delivery. Testing led to the identification of bugs in the system, for example, the slow loading of certain components (e.g., the pregnancy/obstetric history section). The technical partners were able to improve the coding and grouping of questions to speed up the loading of pages. Additionally, as a result of health worker suggestions, the ANC digital module was updated to include the dashboards to support facility based monitoring and reporting of ANC indicators. The integration with existing HMIS systems, such as District Health Information Systems (DHIS) 2.0, also aimed to facilitate data review and aggregation. In Zambia, training materials for supporting the implementation of the ANC digital module were updated based on feedback reported during the FGDs and IDIs.

## Discussion

The formative phase of the NAMAI study aimed to understand and increase acceptability and feasibility of the adapted ANC digital module among potential users in Rwanda and Zambia. The development of the digital ANC module employed a standardized and inclusive process to customize the reference module to Rwandan and Zambian country-adapted ANC packages and local service provision settings. Results were key to responding to user’s needs, refining the module’s design, and identifying bugs and errors in clinical algorithms. User testing was also crucial to ensuring that the ANC digital module addresses the challenges that health worker encounter as they execute their duties. Incorporation of the feedback will increase the likelihood of creating a user-friendly, effective, ANC digital module that may impact healthcare delivery and potentially improve patient outcomes [18].

The efforts described in this manuscript align with established best practices and principles of digital development, notably designing with people and for inclusion, understanding the ecosystem, and anticipating and mitigating potential harms [19]. The user testing process in the adaptation of the reference module to country contexts was an essential step in developing the fifth implementation strategy for the NAMAI study. This process allowed developers to examine and understand the health facility system and context while providing feedback from future users of the customized ANC digital module. Literature has shown that involvement of users in digital health product design is key to ensure that the product is easy to use and fit for purpose [18]. This “co-production” process allowed health workers who provide ANC services to give input into the client flow processes, point of care testing availability, and turnaround of other diagnostic tests. The country-adapted modules were then modified to reflect actual service provision processes at the health facility.

Health worker interaction with the ANC digital modules revealed that it was user friendly and assisted in the delivery of health services. This was highlighted especially in the quick access to longitudinal data for a pregnant woman compared to manual searches in paper registers or patient files. Additionally, the reference module was systematically developed and updated to assist health workers in decision making about appropriate healthcare for specific clinical circumstances [20]. User experience feedback highlighted the appreciation of the decision support mechanism where the ANC digital module flags clinical findings or medical conditions of interest. Also, it provided health workers with clinical knowledge and patient related information that is intelligently filtered or presented at appropriate times to enhance patient care [21].

Users expressed concern that more time was spent on ANC service provision when using the ANC digital module compared to the paper-based ANC registers or patient files/card. This finding aligns with studies which suggest that clinical decision support systems (CDSS) can require more time and effort compared to paper-based methods [22]. However, the timing demands related to using the module are anticipated to reduce with repeat exposure to it. This was especially important as studies have shown that barriers to usability of CDSS include poor integration into practitioner workflow or practitioner non-acceptance of system recommendations [23,24]. While health workers voiced concerns regarding increase workload related to the use of the module, they also suggested the addition of dashboards, and printable patient files and reports for documentation which would improve the ANC digital module’s uptake even with existing staffing challenges. While evidence regarding the impact of digital tools on health worker’s workload is mixed, negative impact can be mitigated with supporting conditions [25,26]. As such, continued leadership from the MoH and local technology partners during the study demonstration phase in line with the WHO SMART guidelines approach will support long-term sustainability and scale-up of digital interventions [13].

Overall, deploying WHO SMART guidelines approach for developing digital tools requires a systematic and iterative process. The strength of our study is the adaptation of the reference module in a standardized manner to develop the Rwandan and Zambian ANC digital modules based on the country-adapted DAKs and service packages. Prior to user testing, the content from the reference module was reviewed and feedback from MOH leadership, including maternal and child programme officers, which led to the modification of some elements to reflect the local ANC service delivery package and systems. The study also had some limitations. This study was conducted in two countries only with small number of health facilities posing limited generalizability. In this formative phase, the tool was also limited to supervisors use and not to the district level, where most data is aggregated, which may have been a challenge in terms of time spent on data entry and aggregation. Another challenge with testing a new tool, however, is that health workers may be influenced, or biased describing the module, given the focus on it during this formative phase. Therefore, the next (demonstration) phase of the NAMAI study will illustrate the use of the ANC digital module during real time service provision. These formative findings will be followed by a rigorous evaluation of the module’s use and assess its effects on key outcomes of interest and costs, including how the Rwandan and Zambian ANC digital modules can support health workers in providing the country-adapted ANC package.

The iterative process in developing country adapted ANC digital modules described in this manuscript is key to their deployments for use during routine ANC provision and proof of concept for the WHO SMART guideline approach. 

## Reflexivity statement

The study author team consisted of maternal and digital health researchers based in Zambia and Rwanda, as well as researchers from different geographic backgrounds working at the global level or in academia. Some of the co-authors are non-practicing clinicians, and another set of authors have obtained ANC services as pregnant women. These experiences as health workers and ANC service-users may implicitly influence our interpretation of the data. We also acknowledge that some of our authors’ positions of working on global policies at the World Health Organization may make us particularly attuned to issues related to feasibility, equity and broader health system implications. This was balanced with perspectives from researchers and implementers working in Zambia and Rwanda to ensure that contextual nuances are not overlooked.

The FGD and IDIs were led by staff from local implementation research partners: Population Council – Zambia and University of Rwanda, entrusted neutral parties, who presented the study and consented the health workers. Interviewers were trained to maintain privacy when recruiting participants ensuring that FGDs and IDIs did not take place in the presence of supervisors and clarifying that refusal to participate would not have implications for performance assessments nor remuneration.
